# BPC 157 in Rodent Ischemia–Reperfusion Injury: A Critical Review of Preclinical Evidence

**DOI:** 10.3390/ijms27188344

**Published:** 2026-09-19

**Authors:** Hüseyin Demirtaş

**Affiliations:** Department of Cardiovascular Surgery, Faculty of Medicine, Gazi University, 06510 Ankara, Türkiye; hdemirtas@gazi.edu.tr; Tel.: +90-5547974981

**Keywords:** BPC 157, ischemia–reperfusion injury, preclinical evidence, rodent models, oxidative stress, nitric oxide, endothelial function, apoptosis, inflammation, VEGF, eNOS, translational pharmacology

## Abstract

Ischemia–reperfusion injury (IRI) is a clinically important consequence of blood-flow restoration after ischemia and contributes to tissue damage across multiple organ systems. This critical review evaluates the preclinical evidence for BPC 157 in rodent IRI and related reperfusion-associated vascular injury models, with particular attention to oxidative stress, endothelial and nitric oxide (NO)-related responses, inflammation, apoptosis, angiogenic signaling, and tissue injury. Searches were performed up to 24 June 2026 across PubMed/MEDLINE, Web of Science Core Collection, Scopus, PMC, publisher/DOI metadata, ClinicalTrials.gov, FDA materials, and WADA materials. Peer-reviewed rodent studies involving conventional organ-specific IRI or reperfusion-associated systemic injury were evaluated as the core IRI/reperfusion evidence according to the experimental model, BPC 157 dose, route and timing, measured outcomes, and methodological limitations, while selected major-vessel occlusion models were considered separately as related in vivo vascular evidence. Endothelial, vascular-ring, human ex vivo, and regulatory/clinical sources were used as contextual evidence rather than as evidence of IRI efficacy. Meta-analysis was not performed because of substantial heterogeneity in organ systems, injury paradigms, doses, routes, treatment timing, and outcome measures. Across the available rodent literature, BPC 157 administration has been associated with attenuation of oxidative injury, modulation of NO-related vascular responses, reductions in inflammatory and apoptotic markers, and changes in VEGF/VEGFR2–Akt–eNOS-related signaling. Organ-specific findings include biochemical, molecular, and histological protection in lower-extremity skeletal-muscle IRI; attenuation of distant-organ injury following limb IRI; neuronal and functional effects in hippocampal IRI; vascular and tissue-protective responses in intestinal/colonic IRI; and hemodynamic and histological effects in hepatic Pringle-maneuver IRI. However, the evidence base remains heterogeneous and frequently relies on short observation periods, single-dose paradigms, and incompletely characterized risk-of-bias domains. Reperfusion-like systemic and major-vessel occlusion models provide additional mechanistic context but should not be considered equivalent to conventional organ-specific IRI. Current evidence therefore supports BPC 157 as a hypothesis-generating investigational candidate for further preclinical IRI research rather than as an established therapy. Independent blinded replication, dose–response and therapeutic-window studies, pharmacokinetic/pharmacodynamic characterization, rigorous toxicology, and ultimately controlled human studies would be required before clinical translation could be considered.

## 1. Introduction

Ischemia–reperfusion injury is a clinically important consequence of restoring blood flow after ischemia. Restoration of blood flow is indispensable for tissue survival, yet reperfusion can intensify the initial ischemic insult through oxidative, inflammatory, endothelial and cell-death mechanisms [1,2,3,4,5]. This problem is clinically relevant in acute limb ischemia, peripheral arterial disease revascularization, tourniquet-associated orthopedic surgery, organ transplantation, resuscitation after shock, myocardial reperfusion and stroke [2,4].

In skeletal muscle, IRI may produce edema, myofiber degeneration, microvascular obstruction, compartment syndrome and systemic inflammatory spillover. Severe lower-limb IRI can also contribute to distant injury in the lung, liver and kidney, indicating that the pathobiology extends beyond the initially ischemic tissue [6].

Most pharmacological approaches to IRI have been designed around single targets, such as antioxidant scavenging, calcium modulation, nitric-oxide donation, mitochondrial-permeability-transition-pore inhibition or cytokine suppression. These mechanisms interact, making single-target interventions difficult to interpret in preclinical IRI models [1,2,3,4,5]. BPC 157 is of interest because the preclinical literature repeatedly describes pleiotropic cytoprotective effects rather than a single isolated mechanism [7,8,9,10,11,12].

The field has recently gained a focused lower-extremity IRI dataset. In a rat model published in Scientific Reports in 2026, BPC 157 was administered intraperitoneally at 20 micrograms/kg at the end of 45 min of ischemia and before 120 min of reperfusion. The study combined serum oxidative biomarkers, gene-expression analysis, immunohistochemistry and histopathology in gastrocnemius muscle [13]. A related Medicina study extended the same conceptual framework to distant-organ injury after lower-extremity IRI [6]. Together with vascular, neurological, intestinal and hepatic models, these studies provide the basis for a focused review of the preclinical evidence and its limitations.

Accordingly, the research question addressed in this review is: what does the available preclinical evidence demonstrate regarding the effects of BPC 157 across rodent organ-specific ischemia–reperfusion and related reperfusion-associated vascular injury models, what biological mechanisms are implicated by these studies, and what methodological and translational limitations currently constrain interpretation of this evidence?

## 2. Methods: Search Strategy, Eligibility and Evidence Grouping

This manuscript is a critical review of preclinical evidence. Studies were organized primarily according to the experimental injury paradigm and affected organ rather than according to their proximity to a single reference model. Conventional organ-specific IRI models were evaluated on their own experimental terms, including lower-extremity skeletal-muscle, hippocampal, intestinal/colonic, and hepatic IRI. Reperfusion-associated systemic injury and major-vessel occlusion models were considered separately because they provide relevant vascular or mechanistic information but do not necessarily reproduce a conventional organ-specific ischemia–reperfusion sequence. Endothelial and vascular mechanistic studies, human ex vivo vascular experiments, and regulatory or clinical sources were retained as contextual evidence and were not treated as rodent IRI efficacy evidence. A meta-analysis was not performed because the included studies differed substantially in model type, dose, route, timing and endpoints.

For each included primary in vivo animal study, data were extracted on species and strain, sex, age or body weight, group size, experimental injury model, duration of ischemia and reperfusion or follow-up, BPC 157 dose, administration route, timing relative to ischemia and reperfusion, chemical form or formulation, vehicle or solvent, preparation conditions, peptide source/manufacturer, stated purity, reported storage or stability information, comparator groups, biological samples or tissues, analytical methods, functional outcomes, reported magnitude and statistical significance of treatment effects, and randomization/blinding procedures. Information not explicitly reported in the original publication was recorded as not reported (NR) rather than inferred from related studies. Because outcome definitions and reporting formats differed substantially among studies, effect estimates were reproduced only when they could be reliably extracted from the original report; otherwise, the direction and statistical significance of the reported effect were summarized without generating post hoc effect sizes.

The review included rodent models exposed to ischemia–reperfusion injury, major-vessel occlusion, decompression/reperfusion or reperfusion-like vascular injury. Eligible studies were assessed for BPC 157 route, timing, dose and endpoints related to redox balance, endothelial function, nitric-oxide homeostasis, inflammation, apoptosis, angiogenic signaling and tissue injury. Vascular-ring/endothelial studies, human ex vivo vascular data, trial-registry entries and regulatory materials were retained only for contextual interpretation.

Searches were performed up to 24 June 2026 across PubMed/MEDLINE, Web of Science Core Collection, Scopus, PMC, publisher pages, DOI metadata, ClinicalTrials.gov, FDA materials and WADA materials. The core search string combined BPC 157 terms with IRI and vascular terms: (“BPC 157” OR “BPC-157” OR “body protection compound 157” OR pentadecapeptide) AND (“ischemia reperfusion” OR “ischemia-reperfusion injury” OR ischemia OR reperfusion OR “vessel occlusion” OR “hindlimb ischemia” OR “lower extremity” OR hippocampal OR intestinal OR hepatic OR myocardial OR “skeletal muscle”) AND (rat OR rats OR rodent OR mice). Mechanistic searches added eNOS, nitric oxide, VEGF, VEGFR2, Akt, Src, caveolin-1, apoptosis, Bax, Bcl-2, caspase, oxidative stress, MDA, SOD, TAS, TOS and inflammation. Because regulatory and clinical-trial information was rapidly evolving, FDA, ClinicalTrials.gov, and WADA sources were additionally rechecked at the time of manuscript revision in September 2026, and the regulatory and trial-status information was updated accordingly.

### 2.1. Eligibility Criteria

For the core IRI/reperfusion evidence synthesis, eligible studies were peer-reviewed original rodent studies in which BPC 157 was administered as an intervention in an experimental paradigm incorporating a defined ischemic or vascular-compromise phase followed by reperfusion, restoration of blood flow, or decompression-associated reperfusion. Eligible studies were required to report extractable histological, biochemical, molecular, physiological, functional, or survival-related outcomes. Permanent major-vessel occlusion models without a defined reperfusion phase were not classified as conventional IRI models; selected studies from this literature were retained separately when they provided directly relevant mechanistic information concerning vascular adaptation, collateral recruitment, or NO-system responses. Endothelial and vascular-ring experiments and human ex vivo vascular studies were likewise retained only as contextual mechanistic evidence. Reviews, conference-only reports, non-peer-reviewed material, anecdotal human use, commercial material, studies without BPC 157, and studies unrelated to ischemia–reperfusion or relevant vascular injury were excluded from the core evidence synthesis.

### 2.2. Study-Selection Process

Records retrieved through the predefined bibliographic searches were consolidated and screened for relevance to BPC 157 and experimental ischemia–reperfusion or closely related vascular injury paradigms. Potentially eligible reports underwent full-text assessment against the predefined eligibility criteria. Six primary in vivo rodent studies met the criteria for the core IRI/reperfusion evidence synthesis [6,13,14,15,16,17]. Studies involving permanent major-vessel occlusion without a conventional reperfusion phase were not counted as core IRI studies; selected reports [18,19] were retained separately as related in vivo vascular evidence because they provided mechanistically relevant information on collateral recruitment, vascular adaptation, and NO-system responses. Endothelial, vascular-ring, and human ex vivo studies [7,8,20] were similarly retained as contextual mechanistic evidence and were not counted as primary IRI studies.

Evidence was interpreted using model-specific rather than anatomically hierarchical criteria. For each rodent study, we considered whether the experimental paradigm represented conventional ischemia followed by reperfusion, a reperfusion-associated systemic injury model, or a major-vessel occlusion model; the affected organ and injury geometry; BPC 157 dose, route, and timing; the biological level of the reported endpoints; duration of follow-up; and the extent of independent replication. Greater caution was applied when model-specific external replication was limited, when randomization or blinding was incompletely reported, when outcomes were limited to biomarkers or histology without functional assessment, or when dose–response and therapeutic-window data were unavailable. Mechanistic vascular studies and translational/regulatory sources were interpreted separately and were not used to increase the certainty of organ-specific IRI efficacy estimates.

Risk of bias in the included in vivo animal studies was formally assessed using SYRCLE’s risk-of-bias tool [21]. Ten domains were evaluated at the study level: sequence generation, baseline comparability, allocation concealment, random housing, blinding of investigators/caregivers, random outcome assessment, blinding of outcome assessors, incomplete outcome data, selective outcome reporting, and other potential sources of bias. Each domain was classified as low, high, or unclear risk of bias on the basis of information explicitly reported in the original publication. A judgment of unclear risk was assigned when reporting was insufficient to support either a low- or high-risk classification; a lack of reporting was therefore not interpreted as evidence that a methodological safeguard had not been implemented.

## 3. Key Mechanisms of Ischemia–Reperfusion Injury

The ischemic phase begins with oxygen and substrate deprivation. Loss of oxidative phosphorylation reduces ATP availability, impairing ion pumps and calcium-handling systems. Intracellular sodium accumulation, reversal of the sodium/calcium exchanger, cytosolic and mitochondrial calcium overload and acidosis destabilize cellular homeostasis. Mitochondria become primed for permeability transition and electron leakage [1,2,3].

Reperfusion then supplies oxygen to injured and metabolically unstable tissue. This generates ROS and RNS through mitochondrial electron transport, xanthine oxidoreductase, NADPH oxidases and uncoupled nitric oxide synthase. Superoxide, hydrogen peroxide, hydroxyl radical and peroxynitrite promote lipid peroxidation, protein oxidation, DNA injury and membrane dysfunction [3,5,22]. In experimental skeletal-muscle IRI, elevated MDA and TOS indicate oxidative injury, whereas reduced SOD and TAS indicate exhaustion of endogenous antioxidant capacity [6,13].

Inflammation amplifies the redox insult. Endothelial activation increases leukocyte adhesion and permeability. Neutrophils infiltrate injured muscle and release proteases, oxidants and cytokines. IL-6, TNF-α and NF-κB-related signaling contribute to local tissue injury and systemic inflammatory propagation [1,2,5]. Apoptosis is coordinated partly through p53 activation, Bax up-regulation, mitochondrial outer-membrane permeabilization and caspase-3 activation; Bcl-2 opposes this process by stabilizing the mitochondrial membrane and shifting the Bax/Bcl-2 balance toward survival [1,4].

Necroptosis, pyroptosis and ferroptosis are increasingly recognized as additional regulated-death mechanisms in IRI, although these pathways remain underexplored in BPC 157-specific lower-limb models [23,24]. ER stress and unfolded-protein-response signaling are biologically relevant to cerebral I/R, but the core BPC 157 lower-limb IRI studies have not directly measured GRP78/BiP, PERK-eIF2α -ATF4/CHOP, XBP1 processing or caspase-12. ER stress is therefore treated here as a future endpoint set rather than current evidence of BPC 157 target engagement [25,26].

## 4. BPC 157: Structural and Pharmacological Rationale

BPC 157 is a synthetic pentadecapeptide with the amino-acid sequence Gly-Glu-Pro-Pro-Pro-Gly-Lys-Pro-Ala-Asp-Asp-Ala-Gly-Leu-Val (GEPPPGKPADDAGLV) and a molecular weight of approximately 1419 Da [9,10,11]. Its reported stability in gastric conditions makes it pharmacologically interesting. However, gastric stability alone does not establish pharmacokinetic suitability, safety or clinical efficacy.

An additional formulation-related feature reported in the broader BPC 157 literature is its administration without a carrier or specialized peptide-delivery system. Sikiric et al. [27] emphasized that BPC 157 has been administered alone across systemic, oral, and local experimental applications, in contrast to several conventional peptidergic or angiogenic growth-factor approaches that may require carrier-based delivery. This characteristic may reduce one potential source of formulation-related confounding; however, it does not eliminate the preparation heterogeneity identified across the primary studies, including differences in chemical form, purity, vehicle reporting, source, and storage conditions.

Recent translational reviews emphasize that BPC 157 still lacks a standardized pharmaceutical-grade formulation, validated human dose selection, complete route-specific pharmacokinetic characterization and a conventional drug-development package [10,11,12,28]. Therefore, the current evidence should be understood as supporting further testing of BPC 157 in rodent IRI models, not as evidence that BPC 157 treats IRI clinically.

## 5. Direct Lower-Extremity IRI Evidence: Gastrocnemius Model

The most directly relevant local skeletal-muscle evidence comes from the 2026 Scientific Reports rat lower-extremity IRI model using abdominal aortic clamping distal to the renal arteries, followed by early reperfusion [13]. This model approximates the reperfusion component of acute limb ischemia or revascularization, but it has important interpretive limits: small groups, male Wistar rats, short reperfusion, serum rather than tissue redox biomarkers and a lack of long-term functional recovery endpoints.

Biochemically, untreated IRI increased MDA and TOS while reducing SOD and TAS. BPC 157 reduced MDA and TOS and restored SOD and TAS toward or beyond control levels [13]. These findings support the hypothesis that BPC 157 limits lipid peroxidation and restores antioxidant capacity. They should not be interpreted as direct proof of radical scavenging alone because BPC 157 may also reduce ROS generation indirectly through microvascular, endothelial or inflammatory effects.

At the gene-expression level, untreated IRI up-regulated *Hif1a*, *Trp53*, *Bax*, *Casp3*, and *Il6*. BPC 157 significantly reduced *Hif1a*, *Trp53*, *Bax*, and *Casp3* expression relative to untreated IRI and increased *Bcl2* expression relative to untreated IRI [13]. The reduction in *Hif1a* expression may indicate attenuation of excessive hypoxia-stress signaling, but it should not be interpreted as proof of direct HIF-1α pathway causality.

Histologically, untreated IRI produced myofiber degeneration, cytoplasmic vacuolization, interstitial edema, neutrophil infiltration, vascular congestion and collagen deposition. BPC 157 improved muscle architecture and reduced Masson-trichrome collagen changes. Immunohistochemistry showed significantly reduced caspase-3 and IL-6 immunoreactivity and partial restoration of VEGF. This protein-level IL-6 reduction should be distinguished from Il6 mRNA expression, where the IRB-to-IR reduction did not reach statistical significance [13].

Importantly, however, these biochemical, molecular, immunohistochemical, and histological improvements were not accompanied by direct assessment of limb function, muscle strength, gait, or longer-term functional recovery; therefore, functional benefit in lower-extremity IRI remains unestablished.

Related evidence from non-IRI gastrocnemius injury models provides additional context for the skeletal-muscle effects of BPC 157. In a rat gastrocnemius crush-injury model, Novinscak et al. [29] reported that locally or intraperitoneally administered BPC 157 was associated with improved macroscopic and histological healing, enzyme-related indices, and functional recovery during follow-up of up to 14 days. In a subsequent gastrocnemius crush-injury study, Pevec et al. [30] reported improved functional, macroscopic, and histological healing with BPC 157, including in animals receiving systemic corticosteroid treatment that impaired muscle healing. These findings are relevant to skeletal-muscle repair and provide functional context beyond the short-term biochemical and histological endpoints available in the lower-extremity IRI literature. However, because crush injury does not reproduce an ischemia–reperfusion sequence, these studies should be regarded as supportive skeletal-muscle healing evidence rather than as direct evidence of efficacy in lower-extremity IRI.

More broadly, a recent review by Matek et al. summarized preclinical evidence for BPC 157 across tendon, ligament, muscle, and associated osteotendinous, myotendinous, and muscle-to-bone junction injuries, including several rodent models with functional and biomechanical outcomes [31]. This broader musculoskeletal literature provides supportive context for the potential of BPC 157 to influence tissue healing and functional recovery beyond the acute biochemical and histological endpoints currently available in lower-extremity IRI. Nevertheless, these heterogeneous musculoskeletal injury models should not be considered direct evidence of efficacy in lower-extremity IRI, and dedicated IRI studies incorporating validated long-term functional endpoints remain necessary.

## 6. From Local Injury to Systemic Injury: Distant-Organ Protection After Limb IRI

Lower-extremity IRI can release inflammatory mediators, oxidized lipids and damage-associated molecular patterns into the systemic circulation. In the lower-extremity IRI companion study, 45 min of limb ischemia followed by 2 h of reperfusion produced distant-organ injury characterized by pulmonary interstitial edema and congestion, hepatic sinusoidal dilation and hepatocellular injury, and renal tubular/glomerular abnormalities. BPC 157 reduced histological injury scores and improved antioxidant indices including TAS, TOS, the oxidative-stress index and PON-1 activity in distant organs [6].

These findings suggest that BPC 157 may affect systemic consequences of limb IRI, although the evidence remains limited to histological and biochemical endpoints. Future work should test circulating cytokines, endothelial-glycocalyx injury, neutrophil extracellular traps, complement activation, mitochondrial-DNA release and organ-specific functional readouts.

## 7. Organ-Specific Rodent IRI and Related Vascular Injury Models

The preclinical BPC 157 literature encompasses several organ-specific rodent IRI models in addition to lower-extremity skeletal-muscle IRI, including hippocampal ischemia–reperfusion following bilateral common-carotid-artery occlusion [20], intestinal/colonic ischemia–reperfusion [15], and hepatic ischemia–reperfusion induced by the Pringle maneuver [16]. These models are considered here as distinct organ-specific IRI paradigms and are evaluated according to their respective injury characteristics, intervention protocols, outcome measures, and methodological limitations rather than according to their anatomical proximity to lower-extremity IRI. Reperfusion-associated systemic injury and major-vessel occlusion models [17,18,19] are considered separately because they provide relevant information on vascular adaptation, collateral recruitment, NO-system responses, and reperfusion-associated injury but do not necessarily reproduce a conventional organ-specific ischemia–reperfusion sequence. The principal rodent IRI and related vascular injury studies are summarized in Table 1A,B.

The hippocampal IRI study provides organ-specific evidence for the effects of BPC 157 in cerebral ischemia–reperfusion and also contributes mechanistic information relevant to vascular and NO-related signaling. BPC 157 was applied during reperfusion after bilateral common-carotid-artery clamping and was associated with functional recovery in memory, locomotion, and coordination assays, reduced hippocampal neuronal injury, and a qRT-PCR expression pattern characterized by increased Egr1, Akt1, Kras, Src, Foxo, Srf, Vegfr2, Nos3, and Nos1, together with decreased Nos2 and Nfkb and no reported Mapk1 activation [14]. These findings are compatible with modulation of vascular, neuronal, and NO-related responses in this cerebral IRI model. However, because the signaling data are predominantly transcript-level observations, they should not be interpreted as evidence of corresponding protein activation or pathway-level causality.

No primary rodent study identified in the present review has evaluated BPC 157 in a conventional coronary-occlusion/reperfusion model of myocardial IRI. The closest cardiac primary evidence is an isoprenaline-induced myocardial-infarction study [32]; however, this model primarily reflects catecholamine-mediated cardiotoxicity rather than a conventional ischemia followed by reperfusion paradigm and was therefore not considered direct myocardial IRI evidence. Broader BPC 157 studies addressing cytoprotection, hemorrhage/thrombosis, musculoskeletal repair, fistula healing, and unilateral adrenalectomy [31,33,34,35] may provide biological or mechanistic context but were not used as evidence of efficacy in organ-specific IRI.

**Table 1:** (**A**). Experimental characteristics and BPC 157 intervention/preparation details of the primary in vivo animal studies. (**B**). Outcome assessment, principal findings, effect magnitude, statistical significance, and methodological safeguards in the primary in vivo animal studies. (**C**). Contextual mechanistic and translational evidence considered separately from the primary rodent IRI efficacy evidence.

**(A)**														
**Study**	**Species/Strain**	**Sex**	**Age/Body Weight**	**Group Size**	**Injury/Ischemia Model**	**Ischemia/Occlusion Duration**	**Reperfusion/Follow-Up**	**BPC 157 Dose**	**Route and Timing**	**Chemical Form/Formulation**	**Vehicle/Solvent and Preparation Conditions**	**Source/Manufacturer**	**Reported Purity**	**Stability/Storage Information**
Demirtas et al. [6]	Rat; Albino Wistar	Male	12 wk; 250–350 g	*n* = 6/group; total *n* = 24	Lower-extremity I/R by infrarenal aortic microclamping; distant lung, kidney, and liver injury	45 min	120 min	20 µg/kg	i.p.; after laparotomy, before aortic clamping (pre-ischemia); BPC-only group: 45 min after laparotomy	BPC 157 peptide; GEPPPGKPADDAGLV; MW 1419.54; specific salt/free-base form NR; no carrier or peptidase inhibitor reported	Control: 0.3 mL 0.9% saline i.p.; peptide reported as soluble in saline/aqueous solution at pH 7.0; detailed reconstitution/preparation procedure NR	Sigma-Aldrich, Taufkirchen, Germany	>95% by HPLC	NR (described as a stable gastric pentadecapeptide, but study-specific storage conditions, in-use stability, and degradation data NR)
Yıldırım et al. [13]	Rat; Albino Wistar	Male	12 wk; 250–350 g	*n* = 6/group; total *n* = 24	Bilateral hindlimb I/R by abdominal aortic clamping distal to renal arteries	45 min	120 min	20 µg/kg	i.p.; at 45th min of ischemia, immediately before reperfusion	BPC 157 peptide; GEPPPGKPADDAGLV; MW 1419.54; specific salt/free-base form NR; no carrier or peptidase inhibitor	SHAM: 0.3 mL 0.9% NaCl i.p.; peptide reported as soluble in saline/aqueous solution at pH 7.0; detailed reconstitution/preparation procedure NR	Sigma-Aldrich, Taufkirchen, Germany	>95% by HPLC	NR (described as a stable gastric pentadecapeptide, but study-specific storage conditions, in-use stability, and degradation data NR)
Vukojevic et al. [14]	Rat; Albino Wistar	Male	12 wk; 200–250 g	≥8/group, endpoint-dependent	Global cerebral/hippocampal I/R by bilateral common-carotid clamping	20 min	qRT-PCR: 1 and 24 h; neurological tests: 24 h; histology: 24 and 72 h	10 µg/kg	Local 1 mL bath to trigonum caroticum; 30 s after clamp release (early reperfusion)	BPC 157 peptide; GEPPPGKPADDAGLV; MW 1419; specific salt/free-base form NR; without carrier or peptidase inhibitor	Freely soluble in water at pH 7.0 and saline; saline used as 1 mL bath control; detailed reconstitution/preparation procedure NR	Diagen, Ljubljana, Slovenia	99% by HPLC; 1-des-Gly peptide reported as main impurity	NR (authors describe BPC 157 as stable; no study-specific storage conditions, in-use stability testing, or degradation profile reported)
Duzel et al. [15]	Rat; Albino Wistar	Male	Age NR; ~200 g	≥6/group	Ischemic colitis: left colic artery/vein ligation producing a 25 mm blood-deprived colon segment; separate late obstruction arm	Main I/R arm: 15 min ligation; late arm: 3 d vascular + bowel obstruction	Main I/R arm: 15 min after ligation removal; late arm followed after ring removal/therapy	10 µg/kg	Local 1 mL bath; 1 min after ligation or 1 min after reperfusion onset; late-treatment arm after established injury	BPC 157 pentadecapeptide; GEPPPGKPADDAGLV; MW 1419; specific salt/free-base form NR	Freely soluble in water at pH 7.0 and saline; equal-volume saline bath control; detailed reconstitution/preparation procedure NR	Diagen, Ljubljana, Slovenia	NR in the primary report	NR
Kolovrat et al. [16]	Rat; Albino Wistar	Male	12 wk; 200 g	*n* = 6/group/interval	Hepatic I/R by Pringle maneuver (portal triad obstruction)	30 min	15 min or 24 h	10 µg/kg or 10 ng/kg	i.p. and local bath; schedules varied by endpoint (pre-occlusion, during occlusion, and immediately after clamp removal/reperfusion)	BPC 157 peptide; GEPPPGKPADDAGLV; MW 1419; specific salt/free-base form NR; without carrier or peptidase inhibitor	Freely soluble in water at pH 7.0 and saline; saline 5 mL/kg control (bath controls commonly 1 mL/rat); detailed reconstitution/preparation procedure NR	Diagen, Ljubljana, Slovenia	99% by HPLC; 1-des-Gly peptide reported as main impurity	NR (authors describe BPC 157 as stable; no study-specific storage conditions, in-use stability testing, or degradation profile reported)
Tepes et al. [17]	Rat; Albino Wistar	Male	12 wk; 200 g	*n* = 6/group/interval	Intra-abdominal hypertension (grade III/IV) followed by decompression/reperfusion; systemic multiorgan injury	25 mmHg/60 min; 30 mmHg/30 min; or 40 mmHg/30 min	60 min; 30 min; or 30 min, respectively	10 µg/kg or 10 ng/kg	s.c.; 3 min after reperfusion/decompression onset	BPC 157 peptide; GEPPPGKPADDAGLV; MW 1419; specific salt/free-base form NR; without carrier or peptidase inhibitor	Freely soluble in water at pH 7.0 and saline; saline 5 mL/kg s.c. control; detailed reconstitution/preparation procedure NR	Diagen, Slovenia	99% by HPLC; 1-des-Gly peptide reported as main impurity	NR (authors describe BPC 157 as stable; no study-specific storage conditions, in-use stability testing, or degradation profile reported)
Vukojevic et al. [18]	Rat; Albino Wistar	Female	12 wk; 200 g	≥6/group, endpoint-dependent	Inferior caval vein ligation up to the right ovarian vein; major venous occlusion/Virchow-triad model	Up to 24 h ligation	No conventional reperfusion phase; early and delayed treatment during ongoing occlusion	10 µg/kg or 10 ng/kg	i.p. and/or local 1 mL bath, endpoint-dependent; early and delayed regimens during ongoing occlusion	BPC 157 peptide; GEPPPGKPADDAGLV; MW 1419; specific salt/free-base form NR	Detailed vehicle/solvent and preparation conditions NR in the accessible primary-text material	Diagen, Slovenia	99% by HPLC; 1-des-Gly peptide reported as main impurity	NR
Amic et al. [19]	Rat; Albino Wistar	Male	Age NR; ~200 g	≥7/group	Superior anterior pancreaticoduodenal vein ligation causing duodenal venous congestion and lesions	Persistent occlusion assessed at 5 min, 30 min, and 24 h	No conventional reperfusion phase	10 µg/kg or 10 ng/kg	Local 1 mL bath; intragastric administration in separate arms; treatment after ligation	BPC 157 pentadecapeptide; GEPPPGKPADDAGLV; MW 1419; specific salt/free-base form NR	Freely soluble in water at pH 7.0 and saline; equal-volume saline controls; detailed reconstitution/preparation procedure NR	Diagen, Ljubljana, Slovenia	NR in the primary report	NR
**(B)**														
**Study**	**Biological Sample/Tissue**		**Analytical Methods**			**Major Functional/Physiological Outcomes**		**Representative Magnitude of Effect**		**Statistical Significance**	**Randomization/Blinding**		**Critical** **Interpretation**	
Demirtas et al. [6]	Lung, kidney, liver tissue		H&E histopathology; tissue TAS, TOS, OSI, PON-1 (commercial spectrophotometric assays)			No organ-specific functional test; distant-organ histological injury and oxidative-status endpoints		Representative: renal PON-1 5.67 ± 0.50 (IR) vs. 7.62 ± 0.70 U/L (IR + BPC); lung PON-1 4.64 ± 0.54 vs. 7.10 ± 0.81 U/L. IR + BPC also lowered TOS/OSI and improved TAS across tissues.		Renal PON-1 *p* = 0.040 vs. IR; lung PON-1 *p* = 0.016 vs. IR; multiple biochemical and histological outcomes *p* < 0.05	Random group allocation reported; sequence-generation method and blinding procedures not clearly reported		Systemic/remote-organ extension of limb I/R; predominantly biochemical and histological outcomes; no long-term functional assessment	
Yıldırım et al. [13]	Serum; right gastrocnemius (histology/IHC); left gastrocnemius (qRT-PCR)		ELISA for MDA/SOD/TOS/TAS; qRT-PCR (Il6, Hif1a, Trp53, Bcl2, Bax, Casp3); H&E/MT histology; IHC/H-score for VEGF, eNOS, IL-6, and caspase-3			No gait/force testing; local muscle architecture, inflammatory infiltration, collagen deposition		MDA 10.02 ± 1.57 → 6.16 ± 1.56 nmol/mL; SOD 14.98 ± 2.33 → 23.74 ± 2.04 ng/mL; TOS 344.30 ± 23.44 → 176.80 ± 84.97 µmol/L; TAS 34.64 ± 4.51 → 53.53 ± 4.65 mmol/L (IR → IR + BPC). Histology injury score reduced.		MDA *p* = 0.0002; SOD *p* < 0.0001; TOS *p* < 0.0001; TAS *p* = 0.0021 vs. IR; histology *p* = 0.0007; additional molecular/IHC comparisons significant as reported	Random assignment reported; histopathology and IHC performed by a pathologist blinded to groups		Most detailed integrated skeletal-muscle I/R dataset; short 2 h reperfusion, single-dose/timing, male-only, no long-term functional outcomes	
Vukojevic et al. [14]	Hippocampus/brain; behavioral performance		Morris water maze; inclined beam walk; lateral push test; H&E neuron counts; RT-qPCR of Vegfr2/Src/NOS/Akt/Kras/Mapk/Srf/Foxo/Nfkb/Egr1 targets			Memory, locomotion, coordination; neuronal survival		BPC 157 maintained preoperative water-maze latency, preserved beam walking and lateral-push resistance, and reduced red-neuron injury at 24/72 h; exact numerical effect sizes are figure-based rather than tabulated in text		Functional and histological differences reported at *p* < 0.05 vs. saline control; gene-expression changes reported at 1 and 24 h	Random assignment reported; observers evaluating experiments/neurological tests blinded to treatment		Direct cerebral I/R evidence with functional outcomes; signaling evidence is transcript-level and does not establish protein activation or pathway causality	
Duzel et al. [15]	Descending colon; colon tissue		USB microcamera vessel mapping; gross pale-area assessment; TBARS/MDA; Griess NO assay; histology			Rapid collateral/arcade-vessel recruitment and restoration of blood supply; mucosal preservation		BPC 157 rapidly increased vessel presentation and normalized MDA/NO; late-treatment arm showed near-preserved mucosa. Exact magnitude varies by experimental arm and is mainly figure/table-based.		Between-group differences considered significant at *p* < 0.05; multiple reported BPC 157 effects significant vs. saline	Animals randomly assigned; assessments performed by observer unaware of treatments		Highly heterogeneous paradigm (ischemia, reperfusion, late obstruction; NO-modulator arms); single local dose and multiple timing contexts limit direct cross-study comparison	
Kolovrat et al. [16]	Liver; GI tract; spleen; heart; blood; portal/caval/aortic circulation		Gross microscopy; H&E histology; MDA/TBARS; AST/ALT/bilirubin; ECG; pressure recordings; thrombosis mass; venography/contrast; vessel presentation			Portal/caval hypertension, aortic hypotension, ECG abnormalities, collateral shunting, thrombosis, ascites, multiorgan injury		Representative: lienal-vein congestion at 15 min reperfusion was 2/2/2 (Min/Med/Max) in controls vs. 1/1/1 with both µg and ng BPC 157 regimens; multiple vascular/lesion scores improved		Representative and multiple endpoint comparisons *p* < 0.05 vs. saline; Fisher exact/ANOVA/Kruskal–Wallis/Mann–Whitney as appropriate	Random assignment reported; experiments assessed by observers unaware of treatment; microscopic injury evaluated by blinded examiner		Broad multi-endpoint study with both µg/ng doses and multiple routes/timings; heterogeneity makes a single pooled treatment estimate inappropriate	
Tepes et al. [17]	Brain, heart, lung, liver, kidney, stomach, small/large intestine, blood; major veins/aorta		Hemodynamic pressure recording; ECG; thrombus mass; USB-microscopy/ImageJ volume assessment; H&E histology; TBARS/MDA			Systemic vascular recovery, intracranial/portal/caval pressure, aortic pressure, arrhythmias, thrombosis, brain swelling, multiorgan injury		BPC 157 markedly attenuated pressure disturbances, thrombosis, organ injury and MDA elevations across all three IAH grades; quantitative magnitude is endpoint- and pressure-grade-specific and presented in multiple original tables		Numerous outcomes *p* < 0.05 vs. saline controls across 10 µg/kg and 10 ng/kg groups	Random assignment reported; experiments assessed by observers blinded to treatment		Reperfusion-associated systemic/decompression model rather than conventional single-organ I/R; extensive outcomes but highly complex injury geometry	
Vukojevic et al. [18]	Plasma; ICV, right ovarian vein, left ovarian vein; vascular/hemodynamic outcomes		Microcamera gross assessment; microscopy; venography; bleeding time; blood pressure; ECG; thermography; MDA/NO in plasma and ICV; gene expression			Collateral bypass recruitment, redistribution of trapped blood volume, venous hypertension, arterial hypotension, tachycardia, thrombosis/bleeding		Accessible primary abstract/preview reports broad attenuation or elimination of ICV-ligation consequences; numerical effect magnitude not reliably extractable from accessible text		Detailed statistical significance NR in accessible abstract/preview	Random assignment reported in accessible article preview; blinding details not reliably extractable		Major-vessel occlusion, not a conventional I/R sequence. Full-text access was unavailable; fields not explicitly verifiable were retained as NR rather than inferred.	
Amic et al. [19]	Duodenum; duodenal tissue; SAPDV/IAPDV/SMV collateral vessels		USB microcamera vessel mapping; gross lesion diameter; histology; TBARS/MDA; Griess NO assay			Collateral vessel recruitment, duodenal perfusion, congestion, mucosal injury		Controls showed vessel branching ≤30% of initial value and lesions >30 mm; BPC 157 commonly increased branching to >60% and few treated rats had lesions >10 mm; NO normalized and MDA remained near normal		Reported comparisons *p* < 0.05 vs. saline for µg/ng regimens across key vascular/lesion outcomes	Random assignment reported; surgery and assessments performed by a blinded observer		Persistent venous-occlusion model without reperfusion; both bath and intragastric regimens and NO-modulator combinations increase heterogeneity	
**(C)**														
**Evidence Source**			**Preparation/Context**			**Main Contribution**				**How Used in this Review**				
Endothelial cells + rat hind-limb ischemia [7]			HUVEC/endothelial culture; rat hind-limb ischemia angiogenesis			VEGFR2 expression/internalization; Akt-eNOS activation; dynasore-sensitive tube formation				Supports an endothelial-response mechanism, but not direct IRI efficacy				
Isolated rat aorta [8]			Aortic rings; vasomotor pharmacology			Endothelium-dependent NO-mediated vasorelaxation via Src-caveolin-1-eNOS; attenuated by L-NAME				Supports vascular-tone mechanism				
Human internal mammary artery ex vivo [20]			Residual IMA rings from CABG; endothelium-intact/denuded			Reduced phenylephrine-induced contraction; greater with intact endothelium; attenuated by NOS inhibition				Provides human ex vivo vascular context, not IRI evidence				
Regulatory and clinical-trial context [36,37,38,39,40]			WADA, FDA, ClinicalTrials.gov			Summarizes safety, approval and trial-readiness boundaries.				Used to limit clinical overstatement; not evidence of IRI efficacy				

Note: (A) NR indicates information not explicitly reported in the original publication. No information on chemical form (including salt/free-base status), formulation, preparation conditions, or stability/storage was inferred from related publications. The authors’ description of BPC 157 as a “stable gastric pentadecapeptide” was not treated as study-specific stability or storage evidence. Reported HPLC purity was reproduced as stated by the original authors and does not establish pharmaceutical equivalence. Abbreviations: HPLC, high-performance liquid chromatography; i.p., intraperitoneal; I/R, ischemia–reperfusion; MW, molecular weight; NR, not reported; s.c., subcutaneous. (B) Effect magnitudes are reproduced only when they could be reliably extracted from the original publication. Where numerical estimates were unavailable or not directly comparable across experimental arms, the direction and statistical significance of the reported effect are summarized without generating post hoc effect sizes. NR, not reported; I/R, ischemia/reperfusion; IHC, immunohistochemistry; MDA, malondialdehyde; SOD, superoxide dismutase; TAS, total antioxidant status; TOS, total oxidant status; OSI, oxidative-stress index; PON-1, paraoxonase-1.

## 8. Mechanistic Evidence: Redox, Endothelium, NO, Apoptosis and VEGF Signaling

**Figure 1:**
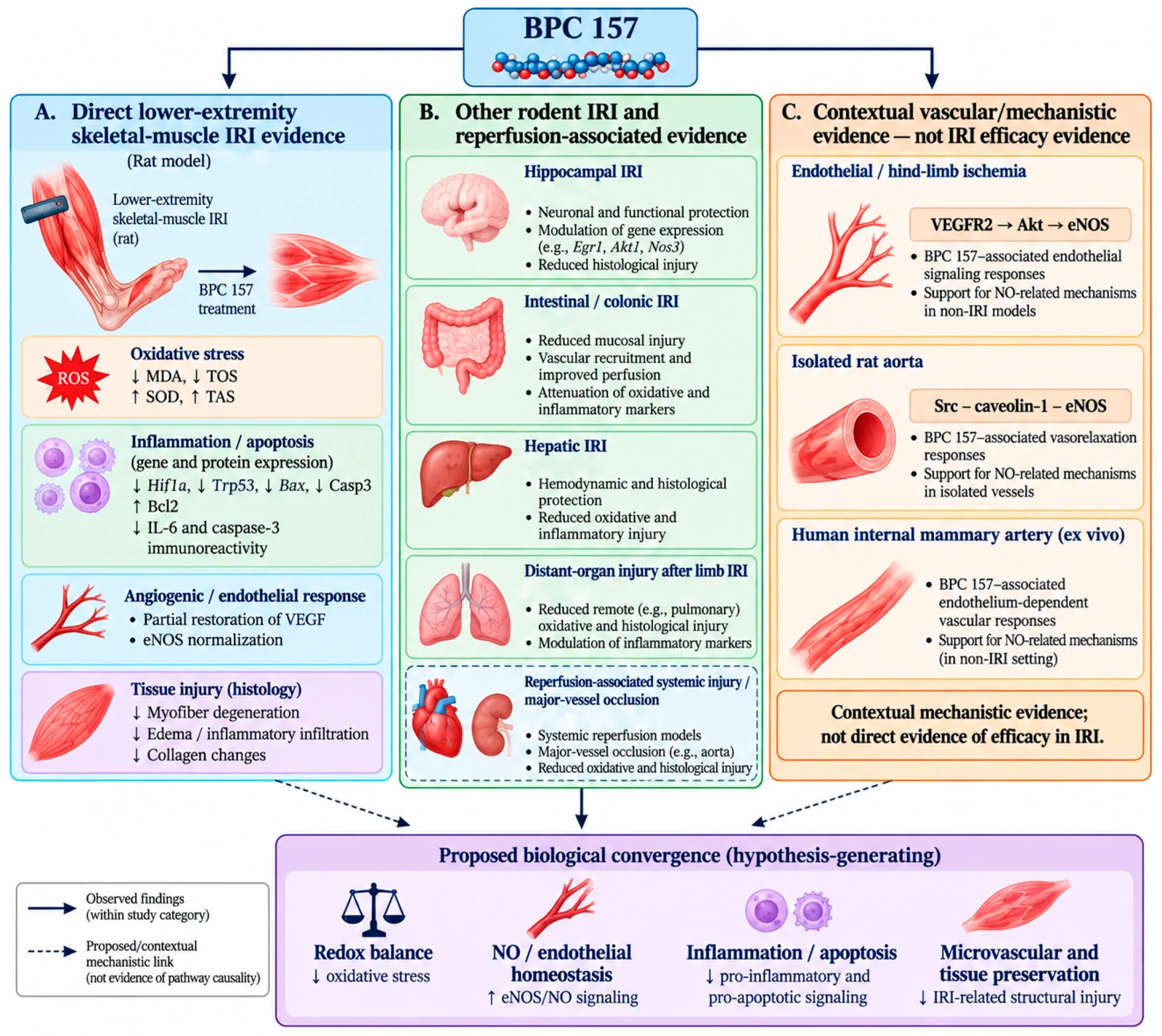
Evidence architecture and proposed mechanistic pathways of BPC 157 across rodent ischemia–reperfusion and related vascular injury models. Direct lower-extremity skeletal-muscle IRI evidence is shown separately from other organ-specific rodent IRI and reperfusion-associated models and from contextual vascular/mechanistic evidence. In lower-extremity skeletal-muscle IRI, BPC 157 was associated with improved redox balance, modulation of inflammatory and apoptotic markers, partial restoration of VEGF-related responses, and reduced histological injury [13]. Other rodent models provide organ-specific or reperfusion-associated evidence involving hippocampal, intestinal/colonic, hepatic, systemic, and major-vessel injury paradigms [6,14,15,16,17,18,19]. Endothelial, vascular-ring, and human ex vivo studies provide contextual support for VEGFR2–Akt–eNOS-, Src–caveolin-1–eNOS-, and NO-related vascular mechanisms [7,8,30] but do not constitute direct evidence of efficacy in IRI. Solid connections indicate findings observed within the respective experimental evidence category, whereas dashed connections indicate proposed or contextual mechanistic relationships. The scheme is intended to illustrate evidence integration rather than establish pathway causality [20,21,22,23,38,39]. ↑ indicates an increase/up-regulation; ↓ indicates a decrease/down-regulation.

The relationship between the principal in vivo IRI evidence and the contextual vascular/mechanistic evidence is summarized schematically in Figure 1, with direct lower-extremity skeletal-muscle IRI, other rodent IRI/reperfusion-associated models, and contextual vascular evidence visually distinguished to avoid conflating mechanistic support with evidence of IRI efficacy. The VEGFR2-Akt-eNOS axis is an important pathway in the contextual mechanistic literature. The principal contextual mechanistic and translational evidence considered in this review is summarized in Table 1C. In endothelial-cell and hind-limb-ischemia models, BPC 157 increased VEGFR2 expression, promoted VEGFR2 internalization and activated downstream Akt-eNOS signaling; dynasore attenuated downstream Akt/eNOS activation and tube formation [7]. These findings support endothelial responsiveness as a possible mechanism, but they are not direct reperfusion-injury evidence.

The NO system represents an additional mechanistic axis relevant to BPC 157. Nitric-oxide biology in IRI is context-dependent [41], and a recent review by Sikiric et al. [27] emphasized a context-dependent interaction between BPC 157 and NO signaling, proposing modulation rather than a uniformly stimulatory or inhibitory effect on NO-related responses. This framework is consistent with experimental observations involving endothelial function, eNOS-related signaling, vascular reactivity, collateral recruitment, and oxidative responses. Physiological NO derived from eNOS contributes to vasodilation, inhibition of platelet aggregation and leukocyte adhesion, and maintenance of microvascular flow, whereas excessive NO production, particularly under inflammatory conditions, may contribute to nitrosative injury through interaction with superoxide and formation of peroxynitrite [41]. Vascular studies suggest that BPC 157 can induce concentration- and endothelium-dependent vasorelaxation through NO-mediated mechanisms and Src–caveolin-1–eNOS signaling in isolated rat aorta [8]. A 2026 human internal-mammary-artery ring study extends this evidence to human arterial tissue, but it remains human ex vivo vascular pharmacology rather than IRI evidence [20]. Nevertheless, these mechanistic associations should not be interpreted as establishing a single causal NO-dependent pathway in IRI, because direct pathway-intervention experiments remain limited and much of the broader NO-related evidence derives from experimental settings outside conventional organ-specific IRI.

The lower-extremity eNOS result should be framed in this broader context. Untreated IRI increased eNOS immunoreactivity, but BPC 157 reduced it toward baseline [13]. In stressed reperfusion tissue, eNOS immunoreactivity does not guarantee coupled NO generation. Future studies should measure NO metabolites, BH4/BH2 balance, 3-nitrotyrosine, phosphorylated eNOS sites, iNOS/nNOS/eNOS separately and microvascular flow rather than treating total eNOS staining as a stand-alone surrogate of beneficial NO signaling.

The p53–Bax–caspase-3 axis is central to the skeletal-muscle findings. At the gene-expression level, untreated IRI increased Trp53, Bax, and Casp3 expression, whereas BPC 157 reduced these pro-apoptotic transcripts and increased Bcl2 expression relative to untreated IRI [13]. BPC 157-associated reductions in IL-6 and caspase-3 immunoreactivity are consistent with reduced inflammatory and apoptotic injury in this model, although the reduction in Il6 mRNA did not reach statistical significance and causal order remains unresolved. Time-course, inhibitor, genetic, receptor-trafficking and exposure–response experiments are required before pathway causality can be claimed.

### Abbreviations and Extraction Notes

BPC 157, Body Protection Compound 157; HPLC, high-performance liquid chromatography; I/R, ischemia–reperfusion; IAH, intra-abdominal hypertension; ICV, inferior caval vein; i.p., intraperitoneal; s.c., subcutaneous; SAPDV, superior anterior pancreaticoduodenal vein; TBARS, thiobarbituric acid-reactive substances; MDA, malondialdehyde; NO, nitric oxide; TAS, total antioxidant status; TOS, total oxidant status; OSI, oxidative-stress index; PON-1, paraoxonase-1; NR, not reported/not reliably extractable. For study [18], the primary full-text PDF was not available; extraction was restricted to the PubMed abstract and accessible publisher preview, and unverifiable fields were marked NR. No unreported manufacturer, vehicle, purity, blinding, or numerical effect estimate was inferred from related publications.

## 9. Evidence Certainty, Independence, and Risk-of-Bias Considerations Across Models

The principal limitations of the current evidence base are its early stage of development, substantial heterogeneity across experimental models, limited independent replication, and incomplete reporting of several risk-of-bias domains. Because the available studies involve distinct organ systems and injury paradigms, evidence certainty was assessed within the context of each model rather than by ranking studies according to their anatomical proximity to lower-extremity IRI. Conventional organ-specific IRI studies provide direct evidence for the respective experimental organ and injury paradigm in which BPC 157 was tested; however, their findings should not be assumed to generalize across organs, ischemia durations, reperfusion intervals, doses, routes, or treatment schedules. Reperfusion-associated systemic injury and major-vessel occlusion models provide complementary vascular and mechanistic information but were interpreted separately from conventional organ-specific IRI models.

A particularly important limitation is the paucity of clinically relevant functional outcome data, especially in lower-extremity skeletal-muscle IRI. The available lower-extremity studies predominantly assessed biochemical, molecular, immunohistochemical, and histopathological endpoints [6,13]. In the direct skeletal-muscle IRI study [13], no gait analysis, muscle-force measurement, limb-use assessment, or longer-term functional recovery endpoint was evaluated, and reperfusion was limited to 120 min. Similarly, the distant-organ study [6] primarily evaluated biochemical and histopathological outcomes rather than organ-specific functional recovery. Consequently, the reported improvements in oxidative-stress markers, inflammatory and apoptotic signaling, and tissue morphology should not be assumed to demonstrate restoration of limb function or durable clinically relevant recovery. Future lower-extremity IRI studies should incorporate validated functional outcomes, including locomotor or gait assessment, muscle strength or contractile-force measurements, limb-use or weight-bearing measures, and longer-term recovery, alongside biochemical and histological endpoints.

### 9.1. Dose Heterogeneity, Administration Timing, Peptide Preparation, and Exposure–Response Uncertainty

A major limitation of the current BPC 157 literature is the pronounced heterogeneity in dosing across experimental models. The primary in vivo studies evaluated in this review used doses ranging from approximately 10 ng/kg to 10–20 µg/kg, corresponding to differences of up to approximately three orders of magnitude. Importantly, these regimens should not be interpreted as demonstrating a broad and reproducible therapeutic dose range. Although beneficial effects have been reported with both ng/kg and µg/kg regimens in some experimental settings, systematic dose–response studies are largely lacking, and comparable efficacy across these dose levels has not been independently replicated across laboratories and organ-specific IRI models. Consequently, the available evidence does not establish a minimum effective dose, an optimal biological dose, a maximal effective dose, or the shape of the dose–response relationship.

Interpretation is further complicated by differences in administration route and treatment timing. BPC 157 has been administered intraperitoneally, subcutaneously, locally as a bath, and intragastrically across the included studies, with treatment delivered before ischemia, during ischemia or vascular occlusion, at the onset of reperfusion, or after reperfusion had begun. Thus, nominal dose comparisons across studies do not represent equivalent exposure comparisons. Local application, in particular, cannot be considered pharmacokinetically equivalent to systemic administration solely on the basis of a dose expressed in µg/kg or ng/kg. Differences in the route, timing, local tissue exposure, absorption, distribution, and peptide stability may therefore contribute to the apparent similarity of biological effects across markedly different nominal doses.

A further limitation is the absence of adequate pharmacokinetic and pharmacodynamic characterization linking the administered doses to systemic or tissue exposure in the IRI models reviewed here. The available studies do not establish plasma or target-tissue concentration–time profiles that would permit direct comparison of exposure after ng/kg versus µg/kg dosing, nor do they define exposure thresholds associated with the reported biochemical, histological, vascular, or functional responses. Accordingly, reports of biological activity at very low doses should be regarded as experimental observations requiring independent confirmation rather than as evidence of an established high-potency exposure–response relationship. Without validated PK/PD data, similar outcomes at widely separated nominal doses cannot by themselves demonstrate dose equivalence or a plateau in pharmacological response.

Peptide preparation represents an additional source of uncertainty when comparing biological effects across studies. Several studies from the same research network reported BPC 157 supplied by Diagen (Ljubljana, Slovenia), in some cases with 99% HPLC purity and 1-des-Gly identified as the principal impurity, whereas the more recent lower-extremity IRI studies used material supplied by Sigma-Aldrich and reported >95% HPLC purity. However, reporting of the chemical form, formulation, vehicle or solvent, preparation conditions, peptide source, stated purity, batch characteristics, storage conditions, stability, and degradation products was incomplete and inconsistent among the primary studies. Where available, these characteristics have been extracted at the study level in Table 1A; information not explicitly reported in the original publication is designated as NR and was not inferred from related reports, including studies from the same research group. This limitation is important because nominally identical BPC 157 doses cannot necessarily be assumed to produce equivalent systemic or target-tissue exposure when the peptide form, purity, formulation, vehicle, preparation procedures, route of administration, or stability differ. Furthermore, the stated HPLC purity alone does not establish pharmaceutical equivalence, chemical stability, or comparable in vivo exposure. Consequently, differences among peptide preparations cannot currently be excluded as contributors to inter-study variability, and these uncertainties reduce confidence in direct cross-study dose comparisons. Future studies should therefore use analytically characterized and standardized peptide preparations with transparent reporting of formulation, purity, preparation procedures, storage conditions, and stability.

Taken together, the apparent activity of BPC 157 across ng/kg and µg/kg doses should not be interpreted as evidence of a well-characterized therapeutic window. Rather, the marked dose heterogeneity, variation in administration route and timing, limited model-specific external replication, inconsistent characterization of peptide preparations, and the lack of model-specific PK/PD data represent major barriers to translational interpretation. Future studies should use pharmaceutical-grade, analytically characterized BPC 157 preparations and prespecified multi-dose designs spanning an appropriate concentration range, with standardized routes and treatment windows. Measurement of plasma and target-tissue exposure, peptide and metabolite stability, and pharmacodynamic responses should be integrated to establish reproducible dose–exposure–response relationships before clinically relevant dose extrapolation is attempted.

### 9.2. Evidence Independence and Model-Specific Certainty

The available BPC 157 studies encompass distinct experimental groups and a range of organ-specific and vascular injury models. Accordingly, the evidence was appraised primarily at the level of each experimental model, considering the study design, injury paradigm, dose, route, treatment timing, outcome measures, duration of follow-up, and methodological reporting. We did not infer a lack of study independence on the basis of author overlap or presumed research-network relationships. Rather, where a specific experimental finding has not yet been reproduced in a separate study using a comparable model and protocol, this is described as limited model-specific external replication. Such limited replication affects the certainty and generalizability of individual findings but does not imply that the original experimental groups were not independent. The model-specific evidence-certainty framework used in this review is summarized in Table 2.

**Table 2:** Model-specific evidence appraisal of BPC 157 in rodent ischemia–reperfusion and related vascular injury models.

Evidence Category/Model	Representative Studies	Principal Strengths	Principal Limitations and Role in Evidence Synthesis
Lower-extremity skeletal-muscle IRI	[13]	Conventional ischemia–reperfusion paradigm with integrated biochemical, molecular, immunohistochemical, and histological assessment of local skeletal-muscle injury.	Provides organ-specific evidence for skeletal-muscle IRI. Interpretation is limited by the short reperfusion interval, single-dose/timing paradigm, limited independent replication, and absence of long-term functional outcomes; findings should not be generalized to other organ-specific IRI models without direct validation.
Distant-organ injury following lower-extremity IRI	[6]	Evaluates systemic consequences of limb IRI across multiple distant organs, combining histopathological assessment with circulating oxidative-stress indices.	Used to identify shared mechanisms; not sufficient to strengthen certainty for lower-extremity IRI. Provides evidence regarding systemic and remote-organ responses following limb IRI rather than independent organ-specific IRI. Interpretation is limited by predominantly histological and biochemical endpoints, absence of organ-specific functional outcomes, and limited independent replication.
Hippocampal IRI	[14]	Conventional cerebral ischemia–reperfusion paradigm incorporating functional, histological, and transcript-level molecular outcomes, including NO- and vascular-signaling-related targets.	Provides organ-specific evidence for cerebral/hippocampal IRI. Mechanistic interpretation is constrained by the predominance of transcript-level signaling data without corresponding protein-level or causal pathway validation; model-specific external replication remains limited.
Intestinal/colonic IRI	[15]	Organ-specific intestinal/colonic ischemia–reperfusion paradigm incorporating vascular recruitment, collateral-circulation, and oxidative/NO-system-related observations.	Provides organ-specific evidence for intestinal/colonic IRI. Heterogeneity in dose, route, and treatment paradigm complicates comparison with other IRI studies and limits definition of a standardized therapeutic regimen; model-specific external replication remains limited.
Hepatic IRI (Pringle maneuver)	[16]	Conventional hepatic inflow-occlusion/reperfusion model incorporating histological, hemodynamic, and collateral vascular responses.	Provides organ-specific evidence for hepatic IRI. Heterogeneous doses and administration routes, together with limited model-specific external replication, restrict estimation of a reproducible treatment effect and preclude direct extrapolation to other organ-specific IRI settings.
Intra-abdominal hypertension/decompression–reperfusion	[17]	Reperfusion-associated systemic injury model incorporating hemodynamic, vascular, oxidative-stress, and multiorgan outcomes following decompression.	Provides evidence regarding systemic vascular adaptation and reperfusion-associated multiorgan injury rather than a conventional single-organ IRI paradigm. Its distinct injury geometry and systemic pathophysiology limit direct comparison with organ-specific IRI models.
Major-vessel venous-occlusion models	[18,19]	Provide in vivo vascular evidence regarding collateral pathway recruitment, adaptation to major venous obstruction, NO-system interactions, and oxidative responses.	These models do not reproduce a conventional ischemia–reperfusion sequence and therefore serve as related vascular and mechanistic evidence rather than organ-specific IRI efficacy evidence. Their findings should not be used to establish efficacy across conventional IRI models.
Mechanistic vascular and endothelial evidence	[7,8,20]	Provides complementary evidence regarding endothelial signaling, VEGFR2–Akt–eNOS-related responses, NO-system modulation, vascular reactivity, and angiogenesis-related mechanisms across cellular, isolated-vessel, in vivo vascular, and human ex vivo settings.	These studies inform biological plausibility and mechanistic interpretation but are not organ-specific rodent IRI efficacy studies. They were therefore not used to increase the certainty of efficacy estimates for individual IRI models.
Translational and regulatory context	[36,37,38,39,40]	Provides information relevant to clinical-development status, regulatory considerations, human evidence gaps, and the translational readiness of BPC 157.	Does not constitute evidence of efficacy in rodent IRI and was considered only when assessing translational readiness, regulatory status, and requirements for future clinical development.

Across models, conclusions were therefore kept cautious when evidence originated predominantly from the same research network; when outcomes were restricted to biochemical markers or histopathology without corresponding functional assessment; when randomization, allocation concealment, blinding, or sample-size justification was incompletely reported; or when BPC 157 was evaluated using a single dose or treatment time point. These limitations were considered within each experimental model and were not used to establish an anatomical hierarchy among organ-specific IRI studies. Study-specific risk-of-bias and reporting-quality considerations are summarized in Table 3A.

**Table 3:** (**A**). Study-specific risk-of-bias and reporting-quality considerations across BPC 157 ischemia–reperfusion and related vascular injury models. (**B**). SYRCLE risk-of-bias assessment of the included in vivo animal studies.

**(A)**										
**Study/Model**			**Main Reporting Concerns**				**Overall Use in this Review**			
Lower-extremity skeletal-muscle IRI [13]			Small cohort, male-only design, short reperfusion, single dose/timing and serum redox biomarkers; blinding details require careful extraction from full text.				Organ-specific skeletal-muscle IRI evidence; requires independent blinded replication.			
Lower-extremity IRI with distant-organ injury [6]			Same model family; histology and biochemical endpoints require clear blinding/outcome-assessment reporting.				Evidence of systemic and remote-organ responses following limb IRI; not sufficient alone to support clinical translation.			
Hippocampal IRI [14]			Behavioral, histological, and qRT-PCR endpoints; the mRNA–protein gap and limited independent replication constrain mechanistic certainty.				Organ-specific hippocampal IRI evidence with complementary information on NO- and vascular-signaling responses.			
Intestinal/colonic and hepatic IRI [15,16]			Protocol heterogeneity and limited model-specific external replication constrain certainty and generalizability.				Organ-specific intestinal/colonic and hepatic IRI evidence, with additional information on vascular and collateral-recruitment responses.			
Intra-abdominal hypertension/reperfusion [17]			Complex systemic model; detection blinding and objective outcome definitions should be verified.				Reperfusion-associated systemic injury evidence; interpreted separately from conventional organ-specific IRI efficacy models.			
Overall animal evidence base			Many risk-of-bias domains remain unclear because sequence generation, allocation concealment, baseline comparability, random housing, blinding, random outcome assessment, and attrition are incompletely reported across several studies.				Conclusions therefore remain cautious because of incomplete methodological reporting and limited model-specific external replication.			
**(B)**										
**Study**	**Sequence** **Generation**	**Baseline** **Comparability**	**Allocation** **Concealment**	**Random** **Housing**	**Blinding of** **Investigators/Caregivers**	**Random Outcome** **Assessment**	**Blinding of** **Outcome** **Assessors**	**Incomplete** **Outcome Data**	**Selective** **Reporting**	**Other** **Bias**
Demirtas et al. [6]	U	L	U	U	U	U	U	L	L	U
Yıldırım et al. [13]	U	L	U	U	U	U	L	L	L	L
Vukojevic et al. [14]	U	L	U	U	U	U	L	U	L	U
Duzel et al. [15]	U	L	U	U	U	U	U	U	U	U
Kolovrat et al. [16]	U	L	U	U	U	U	U	L	U	U
Tepes et al. [17]	U	L	U	U	U	U	L	L	L	U
Vukojevic et al. [18]	U	L	U	U	U	U	U	U	U	U
Amic et al. [19]	U	L	U	U	L	U	L	U	U	U

Risk of bias was assessed at the study level using SYRCLE’s risk-of-bias tool. Each domain was classified as low (L), high (H), or unclear (U) risk of bias on the basis of information explicitly reported in the original publication.

### 9.3. Study-Level Risk-of-Bias Assessment

A structured study-level risk-of-bias assessment was performed using SYRCLE’s risk-of-bias tool [21]. Overall, methodological reporting was incomplete across several domains. Although random assignment was reported in most studies, the method used to generate the allocation sequence was generally not described, and allocation concealment and random housing were rarely reported in sufficient detail. Blinding of outcome assessment was explicitly reported in some studies but remained unclear in others. Accordingly, a substantial proportion of judgments were classified as unclear risk of bias, reflecting insufficient reporting rather than demonstrated methodological inadequacy. No domain was classified as high risk solely because methodological information was absent. The complete domain-level assessment is presented in Table 3B. Against this model-specific evidence framework, the principal molecular targets associated with BPC 157 and the corresponding cautions regarding mechanistic interpretation are summarized in Table 4.

## 10. Translational Barriers, Safety, and Regulatory Context

The main translational limitations of BPC 157 are the lack of a standardized pharmaceutical formulation, limited pharmacokinetic/pharmacodynamic (PK/PD) characterization, insufficiently defined dose–exposure relationships, and the absence of controlled clinical trials in ischemia–reperfusion injury (IRI). Preclinical biological activity does not substitute for pharmaceutical development or establish clinical efficacy. Recent reviews have emphasized unresolved formulation and manufacturing issues, incomplete human pharmacokinetic characterization, possible disconnects between reported biological effects and available exposure data, and very limited controlled clinical evidence [10,11,12,28]. Accordingly, extrapolation from experimental IRI models to human treatment remains premature.

### 10.1. Safety and Toxicological Considerations

The safety profile of BPC 157 remains insufficiently characterized for clinical translation. The absence of a consistent toxicity signal in the preclinical efficacy literature should not be interpreted as evidence of established safety, because most studies reviewed here were designed primarily to evaluate efficacy or mechanistic endpoints rather than to provide comprehensive toxicological assessment. In particular, the available evidence does not adequately define chronic toxicity, reproductive or developmental toxicity, genotoxicity, immunogenicity, carcinogenic potential, or route-specific safety across clinically relevant exposure ranges. Controlled human safety data also remain very limited.

Importantly, potential safety concerns attributable to the BPC 157 peptide itself should be distinguished from risks related to pharmaceutical formulation and product quality. For peptide products, additional concerns may arise from aggregation, peptide-related impurities, degradation products, variable purity or peptide content, formulation components, and inadequate characterization of the active pharmaceutical ingredient (API). These considerations are particularly important when extrapolating from experimentally characterized research preparations to compounded products, which should not be assumed to be pharmaceutically or clinically equivalent. The FDA has specifically identified potential immunogenicity concerns for compounded BPC 157 products administered by certain routes, complexities related to peptide impurities and API characterization, and insufficient safety information to determine whether such products may cause harm in humans [36]. Thus, uncertainty surrounding compounded BPC 157 products should not be attributed solely to the intrinsic toxicity of the peptide; unresolved formulation, purity, manufacturing, and product-quality variables must also be considered.

### 10.2. Regulatory Status and Clinical-Trial Landscape

The regulatory status of BPC 157 should be interpreted separately from questions of experimental efficacy. On 23 July 2026, the FDA Pharmacy Compounding Advisory Committee considered BPC 157 free base and BPC 157 acetate in the context of possible inclusion on the Section 503A Bulks List. This proceeding concerned whether these bulk drug substances should be eligible for use in compounding under Section 503A of the Federal Food, Drug, and Cosmetic Act; it was not a drug-approval proceeding and did not constitute an assessment of BPC 157 efficacy in IRI. The FDA meeting materials identified ulcerative colitis, rather than IRI, as the use evaluated for these BPC 157-related bulk drug substances [37]. Th FDA’s evaluation concluded that the balance of the relevant criteria weighed against placement of BPC 157 free base or BPC 157 acetate on the 503A Bulks List. More broadly, consideration of a substance within the Section 503A compounding framework should not be interpreted as establishing FDA approval, clinical efficacy, safety, pharmaceutical equivalence, or manufacturing equivalence. Compounded drugs are not FDA-approved and are not reviewed by the FDA for safety, effectiveness, or quality before marketing in the same manner as FDA-approved drug products.

The anti-doping status of BPC 157 provides a separate regulatory consideration and should not be conflated with evidence of efficacy or toxicity. The World Anti-Doping Agency (WADA) 2026 Prohibited List includes BPC 157 under class S0 (Non-Approved Substances), which applies to pharmacological substances without current approval by a governmental regulatory health authority for human therapeutic use [38]. This classification is relevant to competitive sport but does not itself establish the efficacy, safety, or toxicological profile of BPC 157.

Registered human studies likewise do not currently provide clinical efficacy evidence for IRI. The earlier Phase I safety/pharmacokinetics study (NCT02637284) remains listed on ClinicalTrials.gov with an unknown recruitment status, with the record last verified in October 2015. Results information has been submitted but is not publicly posted on ClinicalTrials.gov [39]. Moreover, this study was conducted in healthy volunteers rather than in patients with IRI and therefore does not provide clinical efficacy evidence for IRI. A randomized, double-blind, placebo-controlled Phase 2 study evaluating BPC 157 for acute grade II hamstring muscle strain (NCT07437547) is currently registered as recruiting, with no results posted [40]. This trial addresses musculoskeletal injury and repair rather than ischemia–reperfusion injury and therefore cannot be considered evidence of clinical efficacy in IRI.

Taken together, the available regulatory, safety, and clinical evidence supports a cautious translational interpretation. Regulatory consideration within a compounding framework, registration of clinical studies outside the IRI setting, and preclinical biological activity should not be interpreted as evidence that BPC 157 is an approved, clinically effective, or adequately characterized treatment for IRI. Progress toward clinical translation would require standardized pharmaceutical-grade material, rigorous characterization of purity and degradation products, validated bioanalytical methods, route-specific PK/PD studies, comprehensive nonclinical toxicology, and appropriately designed controlled clinical trials.

## 11. Future Research Agenda

Future BPC 157 studies should strengthen the evidence base across organ-specific IRI models while preserving model-appropriate experimental design and outcome assessment. Reperfusion intervals should be extended beyond the predominantly short observation periods used in the current literature to capture early oxidative injury, delayed inflammation, tissue remodeling, and functional recovery. Studies should include both sexes, aged animals, and clinically relevant comorbidity models, including diabetes, atherosclerosis, and peripheral arterial disease where appropriate. Endpoints should extend beyond circulating biomarkers and histopathology to include organ-specific measures of oxidative and nitrosative stress, mitochondrial function, microvascular perfusion, tissue-specific functional recovery, and survival where relevant. In skeletal-muscle IRI specifically, useful endpoints would include local ROS/RNS measurements, mitochondrial respiration, perfusion imaging, capillary density, muscle-force assessment, and gait analysis; corresponding functional and physiological endpoints should be selected for cerebral, intestinal, hepatic, and other organ-specific IRI models. The key requirements for improving the translational readiness of BPC 157 in IRI research are summarized in Table 5.

Mechanistic causality should be tested directly. Candidate approaches include eNOS inhibition, iNOS inhibition, VEGFR2 blockade, Akt inhibition, NF-κB modulation, caspase inhibition, mitochondrial-permeability assays, neutrophil-depletion studies, complement markers and endothelial-glycocalyx injury markers. Studies should follow ARRIVE 2.0 principles, reporting randomization, allocation concealment, blinding, sample-size justification, exclusion criteria and all negative results [42].

ER-stress markers should be treated as future research endpoints rather than as established mechanisms of BPC 157 in IRI. Future organ-specific IRI experiments may incorporate GRP78/BiP, p-eIF2α, ATF4, CHOP/GADD153, spliced XBP1, and caspase-12 time-course measurements, ideally together with causal modulators. These endpoints should be interpreted mechanistically only when measured directly in BPC 157-treated IRI models.

## 12. Conclusions

BPC 157 has a plausible preclinical rationale in rodent ischemia–reperfusion injury because the available studies report effects on oxidative stress, endothelial responses, inflammatory activation, mitochondrial apoptosis, VEGF-related signaling, and NO-system homeostasis. Across organ-specific models, reported findings include biochemical, molecular, histological, functional, and hemodynamic effects in lower-extremity skeletal-muscle, hippocampal, intestinal/colonic, and hepatic IRI. Reperfusion-associated systemic injury and major-vessel occlusion models provide additional vascular and mechanistic context but should be interpreted separately from conventional organ-specific IRI models. Mechanistic vascular studies further support hypotheses involving VEGFR2–Akt–eNOS and Src–caveolin-1–eNOS signaling, although these findings do not establish pathway causality within individual IRI models.

Overall, the current evidence supports a cautious interpretation. BPC 157 is best regarded as an investigational peptide with promising but heterogeneous preclinical findings and low current clinical readiness. Confidence in efficacy remains limited by limited model-specific external replication; heterogeneous experimental paradigms; short observation periods in several studies; incomplete risk-of-bias reporting; and limited dose–response, therapeutic-window, and pharmacokinetic/pharmacodynamic characterization. Independent blinded replication, model-specific dose–response and therapeutic-window studies, pathway-specific causal experiments, pharmaceutical-grade development, GLP-compliant toxicology, and controlled human studies would be required before clinical translation could be considered. Until such evidence is available, BPC 157 should be regarded as a preclinical research candidate requiring independent validation rather than as an established treatment for ischemia–reperfusion injury.

## Figures and Tables

**Table 4:** Proposed molecular targets and cautions for interpretation.

Node	Change in Untreated IRI	BPC 157-Associated Pattern	Caution
MDA/TOS	Increase—lipid peroxidation and oxidant burden	Decrease in lower-limb and distant-organ models [6,13]	Does not prove direct radical scavenging without tissue ROS-source assays.
SOD/TAS/PON-1	Decrease/exhaustion of antioxidant reserve	Restoration or increase [6,13]	Serum and tissue compartments may differ.
p53-Bax-caspase-3	Activation of mitochondrial apoptosis	Down-regulation; reduced caspase-3 staining [13]	Causality requires inhibitor or genetic validation.
Bcl-2	May decrease or be model-dependent	Increased relative to untreated IRI in skeletal-muscle model [13]	The Bax/Bcl-2 ratio may be more informative than either alone.
IL-6/NF-κB	Inflammatory cytokine and transcriptional activation	Lower-limb IRI model: IL-6 immunoreactivity was significantly reduced, while Il-6 mRNA reduction did not reach statistical significance [13]. Hippocampal IRI model: Nos2/Nfkb mRNA down-regulation was reported [14].	Distinguish protein-level immunoreactivity from mRNA expression; Nos2/Nfkb findings come from a brain IRI model and should not be treated as direct lower-limb skeletal-muscle evidence.
VEGF/VEGFR2	Signaling may be impaired by endothelial injury	VEGF partially restored [13]; VEGFR2 internalization and Akt-eNOS activation [7]	Early VEGF signal is not equivalent to mature angiogenesis.
eNOS/Nos3	Protective when coupled; harmful when uncoupled	Model-dependent normalization [13] or activation [8,14,20]	High eNOS staining during IRI may reflect stress rather than effective NO bioavailability.
iNOS/Nos2	Inflammation-driven increase; peroxynitrite risk	Down-regulated in hippocampal IRI model [14]	Needs direct RNS/nitrotyrosine measures in skeletal-muscle IRI.
HIF-1 α	Increase—hypoxic stress and adaptive transcription	IRI-induced increase; BPC 157 significantly reduced Hif-1α expression relative to untreated IRI [13]	This may indicate attenuation of excessive hypoxia-stress signaling, but it does not establish direct HIF-pathway causality or mature angiogenic recovery.
NO-coupling markers	Unclear; eNOS can become uncoupled under oxidative stress	BPC 157-associated eNOS normalization is plausible but not causal [8,13,14,20]	Measure BH4/BH2, NOx, 3-nitrotyrosine and p-eNOS rather than relying on total eNOS staining.

**Table 5:** Translational-readiness checklist for the research agenda.

Requirement	Current Status	Priority for Future Studies
Dose–response and therapeutic window	Insufficient standardization across models	Test ng/kg-to-µg/kg dosing; pre-reperfusion and delayed post-reperfusion schedules.
Pharmaceutical quality	Incomplete formulation/impurity characterization in the public literature [10,11,12]	GMP-grade material; validated purity, stability and degradation assays.
Pharmacokinetics and PK/PD	Possible short plasma exposure with longer biological effects [11]	Measure tissue exposure, metabolites, route-specific bioavailability and exposure–response.
Sex and external validity	Many studies use male rodents only	Include female, aged and comorbidity models such as diabetes and PAD.
Functional outcomes	Histology and biomarkers dominate across several models	Add model-specific functional outcomes, microvascular perfusion imaging, organ-function measures, and survival endpoints where appropriate; include muscle force and gait specifically in skeletal-muscle IRI.
Mechanistic causality	Mostly associative biomarker evidence	Use pathway inhibitors, omics, genetic tools and time-course experiments.
External replication	Model-specific external replication remains limited	Pre-registered, multicenter, blinded replication.
Human translation	No established clinical efficacy for IRI [36,37,38,39,40]	GLP toxicology and Phase I safety before efficacy trials.
Local tissue validation	Several studies rely substantially on circulating or indirect biochemical measures; the skeletal-muscle IRI study included serum MDA/SOD/TAS/TOS measurements	Measure tissue-specific ROS/RNS, mitochondrial respiration, local NO metabolites, and antioxidant-enzyme activity within each organ-specific IRI model.
ER-stress/UPR coverage	Not directly characterized in the organ-specific BPC 157 IRI evidence evaluated in this review	Assess GRP78/BiP, p-eIF2α, ATF4/CHOP, spliced XBP1, and caspase-12 as prespecified future endpoints, ideally using time-course designs and causal modulators.

## Data Availability

No new data were created or analyzed in this study. Data sharing is not applicable to this article.

## Abbreviations

Akt, protein kinase B; Bax, Bcl-2-associated X protein; Bcl-2, B-cell lymphoma 2; BPC 157, Body Protection Compound 157; eNOS/Nos3, endothelial nitric oxide synthase; iNOS/Nos2, inducible nitric oxide synthase; IRI, ischemia–reperfusion injury; MDA, malondialdehyde; NF-κB, nuclear factor kappa-light-chain-enhancer of activated B cells; NO, nitric oxide; PON-1, paraoxonase-1; qRT-PCR, quantitative real-time polymerase chain reaction; RNS, reactive nitrogen species; ROS, reactive oxygen species; SOD, superoxide dismutase; TAS, total antioxidant status; TOS, total oxidant status; VEGF, vascular endothelial growth factor; VEGFR2, vascular endothelial growth factor receptor 2.

## References

[1] Kalogeris T., Baines C.P., Krenz M., Korthuis R.J. (2012). Cell biology of ischemia/reperfusion injury. Int. Rev. Cell Mol. Biol..

[2] Eltzschig H.K., Eckle T. (2011). Ischemia and reperfusion-from mechanism to translation. Nat. Med..

[3] Carden D.L., Granger D.N. (2000). Pathophysiology of ischaemia-reperfusion injury. J. Pathol..

[4] Hausenloy D.J., Yellon D.M. (2013). Myocardial ischemia-reperfusion injury: A neglected therapeutic target. J. Clin. Investig..

[5] Granger D.N., Kvietys P.R. (2015). Reperfusion injury and reactive oxygen species: The evolution of a concept. Redox Biol..

[6] Demirtas H., Ozer A., Yildirim A.K., Dursun A.D., Sezen S.C., Arslan M. (2025). Protective effects of BPC 157 on liver, kidney, and lung distant organ damage in rats with experimental lower-extremity ischemia-reperfusion injury. Medicina.

[7] Hsieh M.J., Liu H.T., Wang C.N., Huang H.Y., Lin Y., Ko Y.S., Wang J.S., Chang V.H.S., Pang J.H.S. (2017). Therapeutic potential of pro-angiogenic BPC157 is associated with VEGFR2 activation and up-regulation. J. Mol. Med..

[8] Hsieh M.J., Lee C.H., Chueh H.Y., Chang G.J., Huang H.Y., Lin Y., Pang J.H.S. (2020). Modulatory effects of BPC 157 on vasomotor tone and the activation of the Src-caveolin-1-endothelial nitric oxide synthase pathway. Sci. Rep..

[9] Seiwerth S., Milavic M., Vukojevic J., Gojkovic S., Krezic I., Vuletic L.B., Pavlov K.H., Petrovic A., Sikiric S., Vranes H. (2021). Stable gastric pentadecapeptide BPC 157 and wound healing. Front. Pharmacol..

[10] Jozwiak M., Bauer M., Kamysz W., Kleczkowska P. (2025). Multifunctionality and possible medical application of the BPC 157 pep-tide-literature and patent review. Pharmaceuticals.

[11] Mateescu D.M., Gavrilescu D.M., Constantinescu F.E., Oancea C., Ilie A.C., Folescu R., Popa M.-D., Iurciuc S., Muresan C.-O., Enache A. (2026). BPC-157 as an investigational peptide therapeutic: Biopharmaceutical challenges, formulation strategies, and translational development barriers. Pharmaceutics.

[12] Yuan C., Demers A., Silva-Ortiz V., Hasoon J.J., Lee W., Dave K., Amirdelfan K., Burke H.W., Christo P.J., Robinson C.L. (2026). From regeneration to analgesia: The role of BPC-157 in tissue repair and pain management. Int. J. Mol. Sci..

[13] Yildirim A.K., Demirtas H., Ozer A., Arslan M. (2026). Protective effects of BPC 157 in rats with experimentally induced lower extremity ischemia-reperfusion injury. Sci. Rep..

[14] Vukojevic J., Vrdoljak B., Malekinusic D., Siroglavić M., Milavić M., Kolenc D., Blagaić A.B., Batelja L., Drmić D., Seiverth S. (2020). The effect of pentadecapeptide BPC 157 on hippocampal ischemia/reperfusion injuries in rats. Brain Behav..

[15] Duzel A., Vlainic J., Antunovic M., Malekinusic D., Vrdoljak B., Samara M., Gojkovic S., Krezic I., Vidovic T., Bilic Z. (2017). Stable gastric pentadecapeptide BPC 157 in the treatment of colitis and ischemia and reperfusion in rats: New insights. World J. Gastroenterol..

[16] Kolovrat M., Gojkovic S., Krezic I., Malekinusic D., Vrdoljak B., Kovac K.K., Kralj T., Drmic D., Barisic I., Pavlov K.H. (2020). Pentadecapeptide BPC 157 resolves Pringle maneuver in rats, both ischemia and reperfusion. World J. Hepatol..

[17] Tepes M., Krezic I., Vranes H., Smoday I.M., Kalogjera L., Zizek H., Vukovic V., Oroz K., Kovac K.K., Madzar Z. (2023). Stable gastric pentadecapeptide BPC 157 therapy: Effect on reperfusion following maintained intra-abdominal hypertension (grade III and IV) in rats. Pharmaceuticals.

[18] Vukojevic J., Siroglavic M., Kasnik K., Kralj T., Stancic D., Kokot A., Kolarić D., Drmić D., Sever A.Z., Barišić I. (2018). Rat inferior caval vein (ICV) ligature and particular new insights with the stable gastric pentadecapeptide BPC 157. Vasc. Pharmacol..

[19] Amic F., Drmic D., Bilic Z., Krezic I., Zizek H., Peklic M., Klicek R., Pajtak A., Amic E., Vidovic T. (2018). Bypassing major venous occlusion and duodenal lesions in rats, and therapy with the stable gastric pentadecapeptide BPC 157, L-NAME and L-arginine. World J. Gastroenterol..

[20] Yildirim A.K., Dastan A.O., Demeli Ertus M., Ensarioglu M., Karabacak K., Pehlivanoglu B. (2026). Endothelium-dependent nitric oxide-mediated vasorelaxant effects of BPC 157 in human internal mammary artery. J. Clin. Med..

[21] Hooijmans C.R., Rovers M.M., de Vries R.B.M., Leenaars M., Ritskes-Hoitinga M., Langendam M.W. (2014). SYRCLE’s risk of bias tool for animal studies. BMC Med. Res. Methodol..

[22] Xiang M., Lu Y., Xin L., Gao J., Shang C., Jiang Z., Lin H., Fang X., Qu Y., Wang Y. (2021). Role of oxidative stress in reperfusion following myocardial ischemia and its treatments. Oxidative Med. Cell. Longev..

[23] Toldo S., Mauro A.G., Cutter Z., Abbate A. (2018). Inflammasome, pyroptosis, and cytokines in myocardial ischemia-reperfusion injury. Am. J. Physiol.-Heart Circ. Physiol..

[24] Zhang M., Liu Q., Meng H., Duan H., Liu X., Wu J., Gao F., Wang S., Tan R., Yuan J. (2024). Ischemia-reperfusion injury: Molecular mechanisms and therapeutic targets. Signal Transduct. Target. Ther..

[25] Nakka V.P., Gusain A., Raghubir R. (2010). Endoplasmic reticulum stress plays critical role in brain damage after cerebral ische-mia/reperfusion in rats. Neurotox. Res..

[26] Wang L., Liu Y., Zhang X., Ye Y., Xiong X., Zhang S., Gu L., Jian Z., Wang H. (2022). Endoplasmic reticulum stress and the unfolded protein response in cerebral ischemia/reperfusion injury. Front. Cell. Neurosci..

[27] Sikiric P., Seiwerth S., Skrtic A., Staresinic M., Strbe S., Vuksic A., Sikiric S., Bekic D., Soldo D., Grizelj B. (2025). Stable Gastric Pentadecapeptide BPC 157 as a Therapy and Safety Key: A Special Beneficial Pleiotropic Effect Controlling and Modulating Angiogenesis and the NO-System. Pharmaceuticals.

[28] McGuire F.P., Martinez R., Lenz A., Skinner L., Cushman D.M. (2025). Regeneration or risk? A narrative review of BPC-157 for musculoskeletal healing. Curr. Rev. Musculoskelet. Med..

[29] Novinscak T., Brcic L., Staresinic M., Jukic I., Radic B., Pevec D., Mise S., Tomasovic S., Brcic I., Banic T. (2008). Gastric pentadecapeptide BPC 157 as an effective therapy for muscle crush injury in the rat. Surg. Today.

[30] Pevec D., Novinscak T., Brcic L., Sipos K., Jukic I., Staresinic M., Mise S., Brcic I., Kolenc D., Klicek R. (2010). Impact of pentadecapeptide BPC 157 on muscle healing impaired by systemic corticosteroid application. Med. Sci. Monit..

[31] Matek D., Matek I., Japjec M., Matek M., Prenc J., Staresinic B., Staresinic E., Prtoric A., Sikiric S., Oreskovic L.B. (2026). Tendon, ligament, and muscle injury, osteotendinous, myotendinous, and muscle-to-bone junction therapy perspectives with growth factors and stable gastric pentadecapeptide BPC 157—A review. Pharmaceuticals.

[32] Barisic I., Balenovic D., Udovicic M., Bardak D., Strinic D., Vlainic J., Vranes H., Smoday I.M., Krezic I., Milavic M. (2022). Stable gastric pentadecapeptide BPC 157 may counteract myocardial infarction induced by isoprenaline in rats. Biomedicines.

[33] Sikiric P., Barisic I., Udovicic M., Bencic M.L., Balenovic D., Strinic D., Posilovic G.Z., Uzun S., Vranes H., Krezic I. (2026). Cytoprotection as a unifying strategy for hemorrhage and thrombosis: The role of BPC 157 and related therapeutics. Pharmaceuticals.

[34] Smoday I.M., Vukovic V., Oroz K., Vranes H., Kalogjera L., Gamulin O., Vlainic J., Milavic M., Sikiric S., Gabaj N.N. (2026). Unilateral adrenalectomy, and the stable pentadecapeptide BPC 157 as therapy in rats-a cytoprotection approach. Pharmaceuticals.

[35] Madzarac G., Becejac T., Penovic T., Drazenovic D., Kralj L., Dolic M.P., Sikiric S., Oreskovic L.B., Oreskovic I., Strbe S. (2026). Tracheocutaneous fistula resolved by pentadecapeptide BPC 157 therapy through the NO-system-triple NO-agent approach in rats. Pharmaceuticals.

[36] U.S. Food and Drug Administration Certain Bulk Drug Substances for Use in Compounding that May Present Significant Safety Risks. https://www.fda.gov/drugs/human-drug-compounding/certain-bulk-drug-substances-use-compounding-may-present-significant-safety-risks.

[37] U.S. Food and Drug Administration July 23–24, 2026: Meeting of the Pharmacy Compounding Advisory Committee. FDA Advisory Committee Calendar. https://www.fda.gov/advisory-committees/advisory-committee-calendar/july-23-24-2026-meeting-pharmacy-compounding-advisory-committee-07232026.

[38] World Anti-Doping Agency The 2026 Prohibited List: World Anti-Doping Code. Valid 1 January 2026. https://www.wada-ama.org/sites/default/files/2025-09/2026list_en_final_clean_september_2025.pdf.

[39] ClinicalTrials.gov NCT02637284: PCO-02—Safety and Pharmacokinetics Trial. NCT02637284.

[40] ClinicalTrials.gov NCT07437547: BPC 157 for Acute Hamstring Muscle Strain Repair (BPC-HAMSTR). NCT07437547.

[41] Lee H.-M., Choi J.W., Choi M.S. (2022). Role of Nitric Oxide and Protein S-Nitrosylation in Ischemia-Reperfusion Injury. Antioxidants.

[42] du Sert N.P., Hurst V., Ahluwalia A., Alam S., Avey M.T., Baker M., Browne W.J., Clark A., Cuthill I.C., Dirnagl U. (2020). The ARRIVE guidelines 2.0: Updated guidelines for reporting animal research. PLoS Biol..

